# Quantification of mitochondrial cfDNA reveals new perspectives for early diagnosis of colorectal cancer

**DOI:** 10.1186/s12885-023-10748-y

**Published:** 2023-03-30

**Authors:** Christian Linke, Richard Hunger, Mark Reinwald, Markus Deckert, René Mantke

**Affiliations:** 1 Department of General and Gastrointestinal Surgery, University Hospital Brandenburg, Brandenburg Medical School Theodor Fontane, Brandenburg, Germany; 2 Department of Hematology, Oncology and Palliative Medicine, University Hospital Brandenburg, Brandenburg Medical School Theodor Fontane, Brandenburg, Germany; 3 Faculty of Health Sciences, Joint Faculty of the Brandenburg University of Technology Cottbus – Senftenberg, the Brandenburg Medical School Theodor Fontane and the University of Potsdam, Potsdam, Germany

**Keywords:** Colorectal cancer, cfDNA, Tumour marker, Predictive model, Diagnostic accuracy

## Abstract

**Background:**

To unravel how the integrity of nuclear and mitochondrial circulating cell-free DNA (cfDNA) contributes to its plasma quantity in colorectal cancer (CRC) patients.

**Methods:**

CfDNA from plasma samples of 80 CRC patients stratified by tumour stage and 50 healthy individuals were extracted. Total cfDNA concentration was determined and equal template concentrations (ETC) were analyzed by quantitative real-time PCR (qPCR) resulting in small and long fragments of KRAS, Alu and MTCO3. The obtained data was also examined relative to the total cfDNA concentration (NTC) and diagnostic accuracy was estimated using receiver operating characteristics.

**Results:**

Total cfDNA levels were significantly higher in CRC group compared to healthy control and increased with tumour stage. Long nuclear fragment levels were significantly lower in CRC patients in ETC but not NTC condition. The integrity indices of nuclear cfDNA decreased from controls to patients with highly malignant tumor. Mitochondrial cfDNA fragment quantities were strongly reduced in early and late stages of tumor patients and prognostic value was higher in ETC. Predictive models based on either ETC or NTC predictor set showed comparable classification performance.

**Conclusion:**

Increased blood cfDNA concentration in late UICC stages inversely correlate with nuclear cfDNA integrity index and suggest that necrotic degradation is not a major cause for higher total cfDNA quantity. The diagnostic and prognostic value of MTCO3 is highly significant in early stages of CRC and can be evaluated more comprehensively, using ETC for qPCR analysis.

**Trial registration:**

The study was registered retrospectively on DRKS, the german register for clinical trials (DRKS00030257, 29/09/2022).

**Supplementary Information:**

The online version contains supplementary material available at 10.1186/s12885-023-10748-y.

## Introduction

Colorectal cancer (CRC) is among the three most common diagnosed cancer and cause of death in developed countries [1]. The risk of developing colorectal cancer increases with age and its malignant progression lasts about 10—15 years, thus offering time for an early diagnosis [2]. The survival chances for cancer patients also depend strongly on the disease stage and can be significantly improved by early detection [3].

In industrialized countries, screening for CRC is initially made using guaiac-based fecal occult blood tests (gFOBT) or haemoglobin-based fecal immunological tests (FIT). Subsequently, CRC can be detected and monitored endoscopically (colonoscopy) which represents the gold standard method for CRC examination, but lacks a comprehensive application in routine diagnostics [4]. The use of fecal-based tests such as gFOBT or FIT are widely criticized among clinical experts, since the average sensitivity of stool blood tests are substantially limited [5, 6]. In addition, false-positive test results can be obtained due to cancer-unspecific internal bleedings, thus leading to potentially unnecessary follow-up examinations [7].

In order to improve the early detection and survival of CRC, highly sensitive, low-priced diagnostic tools should be developed. Currently, the molecular genetic analysis of circulating cell-free DNA (cfDNA) in the bloodstream of patients (“liquid biopsy”) has proven to be a promising approach [8]. CfDNA fragments are released from normal and tumor cells as a result of cellular degradation processes or exocytosis [9, 10]. In multiple cancer entities, including CRC, the concentration of cfDNA in patient plasma was found to be significantly higher compared to healthy control subjects [11]. Several studies demonstrated that the sensitivity and specificity of cfDNA quantification is superior compared to gFOBT, reviewed in Petit et al. [12].

A further improvement of cfDNA-based diagnostics was proposed determining the length of the cfDNA fragments [12–14]. This approach comprises the amplification of a short (~ 100 bp) and a long fragment (~ 250 bp) of cfDNA markers via quantitative real-time PCR (qPCR). The ratio between long and short fragments forms the so-called DNA integrity index (DII) and is believed to represent the difference between apoptotic and necrotic cell degradation processes. It has been shown that as a result of necrosis, genomic DNA fragments with a length of > 250 bp are formed, whereas apoptotic nuclease activity results in a fragment length of < 180 bp [15]. It is assumed that cancer cells presumably initiate necrotic cell death due to an active suppression of p53-mediated apoptosis [16]. For CRC, some studies reported an increased DII [13, 14, 17–20], whereas others presented a different view, challenging the hypothesis of elevated necrotic cell death associated with increased cfDNA levels in the plasma of tumor patients [21–27].

In addition to nuclear cfDNA (n-cfDNA) analysis, quantification of cell-free mitochondrial DNA (mt-cfDNA) in blood plasma was shown to improve early detection of CRC [21, 28], presumably due to higher copy number per cell [29, 30]. Currently, research on the association between an altered mt-cfDNA concentration and presence of CRC resulted in unclear data. A systematic analysis of DNA integrity index with nuclear and mitochondrial markers in distinct tumour stages of CRC has not been published yet.

The aim of this study was, first, to analyze total cfDNA concentration in CRC patients with different histopathological stages. Second, to quantify fragments of well-known n-cfDNA markers KRAS and Alu, as well as mt-cfDNA marker MTCO3 independent from total cfDNA levels and third, to examine the integrity index. We also assessed whether our data can be compared to previous work, that measured these markers as a function of total cfDNA concentration. Finally, we evaluate the diagnostic accuracy of our approach comparing the discriminative ability of all markers and their ratios (a) independent (“equal template concentration”—ETC) and (b) in relation to the total cfDNA concentration (“normalized to total cfDNA”—NTC).

## Material and methods

### Patients and samples

The study was conducted in accordance with the Declaration of Helsinki and performed under STARD guidelines [31]. The study is officially registered on DRKS, the german register for clinical trials (DRKS00030257). Local ethics committee approval and informed patient consent was obtained. All colorectal cancer patients were ≥ 18 years of age and had not been treated with radiotherapy or chemotherapy prior to blood sampling. Blood samples of 80 consecutive patients were collected before surgical care. The CRC cohort included patients with UICC stage I (*n* = 21), UICC II (*n* = 21), UICC III (*n* = 20) and UICC IV (*n* = 18), confirmed after surgery by histopathological examination according to established standard diagnostic procedures. Blood samples from 50 healthy individuals were provided by Central BioHub®, a commercial Biobank that hosts collections of human biospecimen for scientific research.

All blood samples were prospectively collected using K2EDTA BD Vacutainer® Collection Tube (Becton Dickinson, Germany). On the day of venipuncture, plasma samples were centrifuged at 2,000 × g for 10 min and the supernatants were carefully removed, avoiding the buffy-coat. Plasma aliquots of CRC patients and controls were stored at –80 °C until analysis.

### Sample processing, total cfDNA extraction and quantification

A total of 1 ml of blinded plasma samples from all individuals were thawed at room temperature and centrifuged at 16,000 × g for 10 min at 4 °C. The supernatant was transferred to 1.5 ml tube and cfDNA extraction performed using the QIAamp® Circulating Nucleic Acid Kit (Qiagen, Germany) according to the manufacturer´s protocol, except the column-based isolation of total cfDNA, that was performed with centrifugation at 2,000 × g instead of using the vacuum pump. Extracted cfDNA was eluted with 50 µl elution buffer and total cfDNA concentration was determined using Qubit™ dsDNA HS Assay Kit and Qubit™ 3.0 Fluorometer (ThermoFisher, Fisher Scientific, Invitrogen, Germany). The samples were stored at –20 °C prior to quantitative real-time PCR (qPCR) analysis.

### Quantity and integrity index of nuclear and mitochondrial cfDNA

To measure n-cfDNA and mt-cfDNA marker quantities, short and long fragments of KRAS, Alu and MTCO3 markers were targeted with qPCR. Note, that n-cfDNA markers in the plasma of healthy individuals and CRC patients were analysed independent from their total cfDNA using equal template concentrations (ETC). Technically, we believed that this experimental approach ensures equal molarity of components in qPCR reactions, and therefore may help to precisely evaluate, whether short and long cfDNA fragments are either increased, decreased or unaltered in both groups.

QPCR was performed on a Light-cycler 96 (Roche, Germany). All cfDNA samples were diluted to a final template concentration of 0.1 ng/µl and 2 µl used as template. All qPCR reactions were performed with 15 µl reaction volume containing 1 × PowerUp™ SYBR™ Green Master Mix and 0.25 µM of primer. Human genomic DNA isolated from pancreatic tissue was used as positive and a no template control as negative control. Cycling conditions consisted of initial denaturation at 95 °C for 2 min, and 40 cycles of of 95 °C for 15 s and 60 °C for 1 min. A standard curve with serial dilutions of genomic DNA (0.005, 0.01, 0.025 0.05, 0.5 1.0, 2.0 ng/µl) was used to calculate a logarithmic trend line and cycle threshold (Ct) values of measured qPCR quantities returned along the trend line. Resulting data (ng/µl) were used to determine plasma cfDNA concentration as follows: Plasma cfDNA concentration = qPCR data (ng/µl) × extraction elution volume (µl) ÷ plasma volume (µl). The final data is expressed in ng/ml using the mean values of qPCR triplicates (ETC).

To compare our results with existing data from literature, we calculated the absolute cfDNA quantity of short and long cfDNA fragments by normalizing the data with the dilution factor of each sample used to obtain 0.1 ng/µl template concentration (normalized to total concentration—NTC).

The DNA integrity index was calculated as the ratio of long to short fragments (e.g. KRAS 305/KRAS 67). Oligonucleotides of KRAS 67, KRAS 305, Alu 115, Alu 247, MTCO3 67 and MTCO3 296 are depicted in Table S4.

### Statistical analysis

First, the group of healthy individuals were compared with the entire CRC cohort, and second, the CRC cohort was subdivided by UICC stage and compared with each other and with the control group. The case numbers are sufficient to differentiate healthy individuals from total CRC patients with medium effect sizes with sufficient statistical power (80%) with regard to the analysed cfDNA markers.

Data was analysed in three steps. First, total cfDNA concentration, short and long fragment quantities and DII scores were summarized descriptively by median and interquartile range (IQR) and graphically presented by boxplots. Additionally, average biomarker levels were compared between analysis groups by Mann–Whitney U-Tests (complete CRC cohort vs. controls) and Kruskal–Wallis-Tests (controls vs. CRC cohort stratified by UICC stage), followed by Bonferroni-Holm-adjusted multiple pairwise comparisons in case of significant omnibus test results.

Second, diagnostic accuracy was assessed separately for each biomarker (stratified by ETC/NTC condition) regarding the ability to distinguish between healthy and CRC cohort individuals and individuals of the several UICC stages, respectively. To keep the number of comparisons manageable, individuals with UICC stages I and II, and stages III and IV were each grouped together for further analyses. Optimal cut-off values were determined using the Youden’s index. To quantify diagnostic accuracy sensitivity, specificity, positive predictive value (PPV), negative predictive value (NPV) and AUC with 95% confidence limits (CI) were calculated.

To assess the combined performance of the biomarkers to differentiate between healthy individuals and individuals with CRC, as well as patients with different UICC stages, a relaxed multinomial logistic LASSO (least absolute shrinkage and selection operator) regression model with tenfold cross-validation to select optimal penalty was performed. In this approach, a penalty term in the regression equation reduces the absolute value of the regression coefficients (possibly to zero) and therefore regulates the impact that a single predictor may have on the overall regression (or inclusion at all) [32, 33]. During model fitting identifies two important penalty values, one that minimizes the misclassification error and one that represents a stronger penalty that is still within one standard error of the minimum misclassification error. For the reported models, we used the higher penalty value, because it results in a sfimaller number of selected predictor variables and thus more externally valid models. The relaxation undoes the shrinkage of the regression coefficients (unpenalized regression) of those predictor variables with regression coefficients greater than zero. Estimated multinomial logit coefficients were exponentiated and reported as relative risk ratios.

This approach offers some important advantages over classical regression analysis: 1) the selected predictors (corresponding regression coefficients are greater than zero) are more robust for future predictions, as this approach is less susceptible to random noise in the predictor values; 2) problematically high intercorrelations between the predictor values (multicollinearity) can be adequately taken into account; 3) overfitting to the data is avoided.

Diagnostic accuracy performance in predicting disease status was assessed using all biomarkers of the ETC condition or the NTC condition and a combined set of markers of both conditions, with healthy individuals as reference category and individuals with UICC stages I/II and III/IV each collapsed in one category.

Diagnostic accuracy performance in predicting disease status was assessed using all biomarkers of the ETC condition or the NTC condition. Data analysis was performed with R version 4.2.1 (R Software Foundation, Vienna, 2022), especially utilizing the “epiR” package (version 2.0.50) to calculate diagnostic accuracy values, “pROC” (version 1.18.0) to calculate and display ROC-curves [34], and “glmnet” (version 4.1–4) to fit logistic LASSO regression models [32].

## Results

### Patient characteristics

The study cohort comprised 80 CRC patients (47 males; median age: 68 years, IQR: 61—75) and 50 self-reported healthy individuals (29 males; median age: 57 years, IQR: 55—66) as controls. UICC staging of the CRC cohort and further demographic characteristics are summarized in Table 1.

**Table 1:** Patient characteristics and cfDNA concentrations in study groups

Characteristic	by study group			by UICC stage				
	Controls, *N* = 50	CRC patients, *N* = 80	*P*-value	UICC I, *N* = 21	UICC II, *N* = 21	UICC III, *N* = 20	UICC IV, *N* = 18	*P*-value
Gender			0.9327					0.3036
female	21 (42%)	33 (41%)		12 (57%)	6 (29%)	8 (40%)	7 (39%)	
male	29 (58%)	47 (59%)		9 (43%)	15 (71%)	12 (60%)	11 (61%)	
Age	57.0 (55.0, 66.0)	68.0 (61.0, 75.2)	< 0.0001	66.0 (61.0, 73.0)	72.0 (63.0, 76.0)	64.5 (58.5, 74.0)	69.5 (63.5, 73.5)	0.6123
Total cfDNA (ng/ml)	6.10 (4.50, 8.28)	10.51 (6.92, 19.83)	< 0.0001	8.87 (6.75, 12.60)	9.09 (6.17, 14.22)	12.46 (7.36, 20.71)	20.66 (9.74, 37.24)	0.0084

Reported values are frequencies (percent) and medians (IQR) and test results of group comparisons

*CRC* Colorectal cancer, *UICC* Union internationale contre le cancer, *IQR* Interquartile range

Generally, increased age was associated with higher total cfDNA fragment concentrations (*r*_*S*_ = 0.31; *P*_*adjusted*_ = 0.008) in the complete sample analysis. Significant correlations between age and cfDNA fragment quantities measured at ETC were primarily observed in the complete sample analysis (Supplementary Table S1). With a few exceptions, further stratification by disease status or UICC stage resulted in a drop to non-significant associations. Normalized cfDNA markers (NTC) were not correlated with age, except the DII values (all *P*’s ≤ 0.021; Supplementary Table S2). Subgroup analyses stratified by UICC stage showed only occasional significant correlations with age.

### Quantification of total cfDNA

**Fig. 1:**
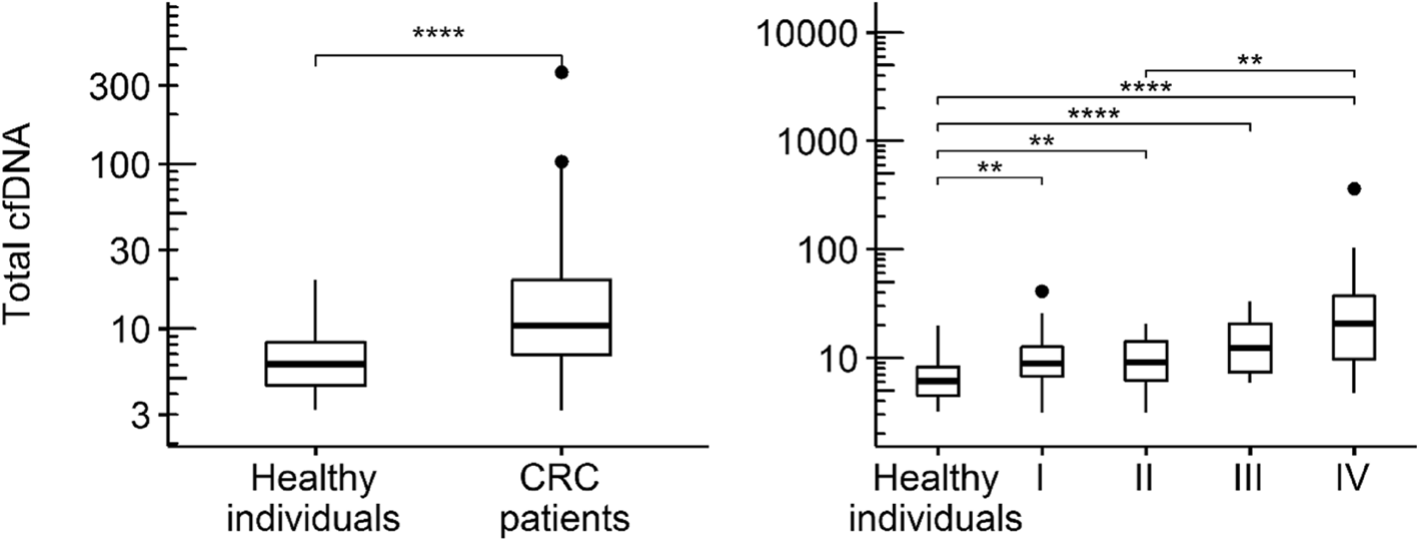
Boxplots of total cfDNA concentrations in healthy individuals, the CRC cohort and CRC patients stratified by UICC stage. Note. Boxplots present total cfDNA concentration (in ng/ml) by study group (left panel) and further subdivided by UICC stage (right panel). Roman numerals on x-axis ticks refer to UICC stage. Stars indicate significant group differences in multiple Mann–Whitney-Tests (Bonferroni-Holm-adjusted in right panel): * … *P* ≤ 0.05, ** … *P* ≤ 0.01, *** … *P* ≤ 0.001, **** … *P* ≤ 0.0001. CRC: colorectal cancer; UICC: Union internationale contre le cancer

Total cfDNA extracted from plasma of all individuals was measured using the PicoGreen method, resulting in a median concentration of 8.39 ng/ml in all samples (IQR: 4.57 – 8.35, range: 3.15 – 362 ng/ml). Compared to healthy controls, CRC patients had significantly raised levels of total cfDNA (*P* < 0.0001), with a median (IQR) of 10.75 (6.86 – 19.87) compared to 6.10 (4.50 – 8.28). Comparison of controls with CRC-cohort of different pathological grades showed significantly raised levels of total cfDNA (Kruskal–Wallis-test: *P* = 0.0084) for UICC-I (*P* = 0.0020), UICC-II (*P* = 0.0030), UICC-III (*P* < 0.0001) and UICC-IV (*P* < 0.0001). Specifically, the median (IQR) in stage I was 8.87 (6.75 – 12.60) ng/ml, stage II 9.09 (6.17 – 14.22), stage III 12.47 (7.36 – 20.71) and stage IV 20.66 (9.74 – 37.24), respectively (Table 1 and Fig. 1).

### Nuclear and mitochondrial cfDNA in CRC patients versus healthy individuals

**Fig. 2:**
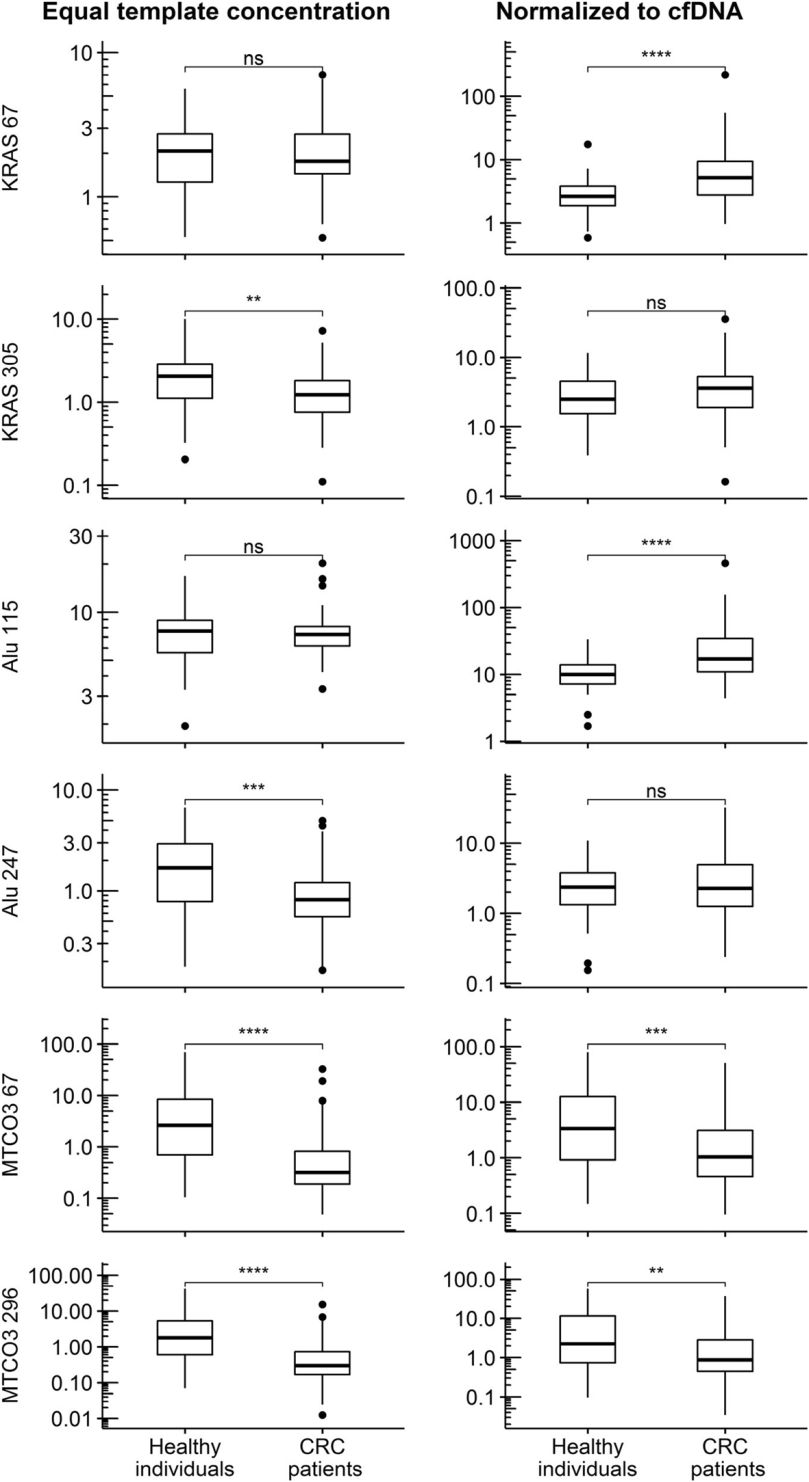
Boxplots of n-cfDNA and mt-cfDNA marker concentrations by study group. Note. Boxplots present marker concentrations (in ng/ml) of qPCR analysis using equal template concentrations (left panel) and normalized to total cfDNA concentration (right panel). Stars indicate significant group differences by Mann–Whitney-Test: * … *P* ≤ 0.05, ** … *P* ≤ 0.01, *** … *P* ≤ 0.001, **** … *P* ≤ 0.0001. CRC: colorectal cancer

Median and IQR values of studied cfDNA markers are summarized by study group in Table 2 (left panel) and Fig. 2. At ETC, no significant differences between healthy individuals and CRC patients were found in the short nuclear cfDNA fragments KRAS 67 and Alu 115. However, for both long fragments (KRAS 305 and Alu 247) significantly decreased concentrations in the CRC cohort were observed (*P* = 0.0021 and *P* = 0.0003, respectively). Conversely, short but not long fragmented markers showed significant differences between controls and CRC patients (each *P* < 0.0001) in NTC condition.

**Table 2:** Data of nuclear and mitochondrial cfDNA markers in healthy individuals, the CRC cohort and CRC patients stratified by UICC stage

	by study group			by UICC stage				
	Healthy individuals, *N* = 50	CRC patients, *N* = 80	*P*	UICC I, *N* = 21	UICC II, *N* = 21	UICC III, *N* = 20	UICC IV, *N* = 18	*P*
Equal template concentration (ETC)								
KRAS 67	2.08 (1.27, 2.72)	1.77 (1.45, 2.72)	0.7979	2.11 (1.64, 3.24)	1.70 (1.43, 2.41)	1.65 (1.39, 2.33)	2.43 (1.41, 2.76)	0.1836
KRAS 305	2.06 (1.11, 2.86)	1.23 (0.76, 1.81)	**0.0021**	1.78 (1.12, 2.06)	1.35 (1.01, 1.87)	1.17 (0.74, 2.46)	0.74 (0.46, 1.17)	**0.0037**
Alu 115	7.64 (5.59, 8.89)	7.26 (6.18, 8.15)	0.4104	7.26 (6.89, 7.61)	7.67 (6.32, 8.36)	6.42 (5.33, 7.88)	6.99 (6.01, 8.07)	0.2771
Alu 247	1.70 (0.78, 2.92)	0.82 (0.56, 1.21)	**0.0003**	1.06 (0.72, 1.62)	0.74 (0.58, 1.54)	0.91 (0.49, 1.17)	0.76 (0.45, 1.13)	0.5301
MTCO3 67	2.61 (0.70, 8.45)	0.32 (0.19, 0.82)	** < 0.0001**	0.51 (0.16, 0.79)	0.27 (0.17, 0.41)	0.91 (0.27, 4.09)	0.24 (0.20, 0.44)	**0.0406**
MTCO3 296	1.82 (0.60, 5.28)	0.30 (0.17, 0.73)	** < 0.0001**	0.36 (0.23, 0.78)	0.22 (0.17, 0.38)	0.43 (0.22, 4.13)	0.24 (0.14, 0.49)	0.0896
Normalized to total cfDNA (NTC)								
KRAS 67	2.63 (1.86, 3.85)	5.24 (2.76, 9.34)	** < 0.0001**	4.89 (3.21, 7.58)	4.58 (1.89, 5.88)	4.85 (3.29, 9.13)	12.08 (4.70, 21.31)	**0.0099**
KRAS 305	2.50 (1.55, 4.52)	3.61 (1.90, 5.27)	0.0500	3.83 (1.90, 4.33)	2.57 (1.92, 3.83)	4.27 (1.99, 5.27)	5.14 (2.03, 7.81)	0.2609
Alu 115	9.98 (7.19, 13.86)	17.01 (10.91, 34.60)	** < 0.0001**	14.67 (10.45, 27.50)	14.30 (9.07, 23.41)	18.19 (10.76, 32.48)	36.64 (16.53, 67.65)	**0.0212**
Alu 247	2.37 (1.32, 3.82)	2.28 (1.25, 4.94)	0.7036	2.34 (1.29, 3.55)	1.70 (0.99, 3.72)	2.34 (1.31, 4.32)	3.54 (1.80, 8.85)	0.2047
MTCO3 67	3.36 (0.92, 12.64)	1.04 (0.46, 3.10)	**0.0008**	0.83 (0.45, 2.84)	0.65 (0.39, 0.83)	2.37 (0.62, 9.20)	1.23 (1.03, 2.71)	**0.0089**
MTCO3 296	2.25 (0.73, 11.51)	0.87 (0.44, 2.85)	**0.0019**	0.89 (0.47, 3.05)	0.47 (0.31, 0.85)	1.55 (0.54, 8.13)	1.31 (0.56, 1.81)	**0.0334**
DNA integrity index (DII)								
Index KRAS 305/67	1.05 (0.73, 1.46)	0.63 (0.44, 1.02)	**0.0003**	0.81 (0.50, 1.09)	0.74 (0.54, 1.08)	0.69 (0.53, 1.30)	0.38 (0.22, 0.53)	**0.0013**
Index Alu 247/115	0.22 (0.14, 0.33)	0.12 (0.08, 0.18)	** < 0.0001**	0.12 (0.09, 0.23)	0.10 (0.09, 0.17)	0.13 (0.10, 0.18)	0.09 (0.06, 0.14)	0.4174
Index MTCO3 296/67	0.60 (0.46, 1.04)	1.00 (0.81, 1.10)	**0.0046**	1.07 (0.95, 1.21)	0.97 (0.86, 1.06)	0.99 (0.74, 1.07)	0.98 (0.61, 1.17)	0.2515

Reported are median (IQR) values and results of the group comparisons by Mann–Whitney-U-test (healthy individuals vs CRC cohort) and Kruskal–Wallis-Test (UICC stages). Concentrations (in ng/ml) in healthy individuals and CRC patients measured by qPCR using equal amounts of template and subsequently normalized to their respective total cfDNA concentration

*CRC* Colorectal cancer, *IQR* Interquartile range, *UICC* Union internationale contre le cancer

The comparison of mt-cfDNA fragment concentrations (MTCO3) between healthy individuals and CRC patients yielded highly significantly decreased values in the CRC cohort in both the ETC and NTC condition. The group differences are slightly more pronounced in the ETC condition.

### Nuclear and mitochondrial cfDNA in different UICC stages

**Fig. 3:**
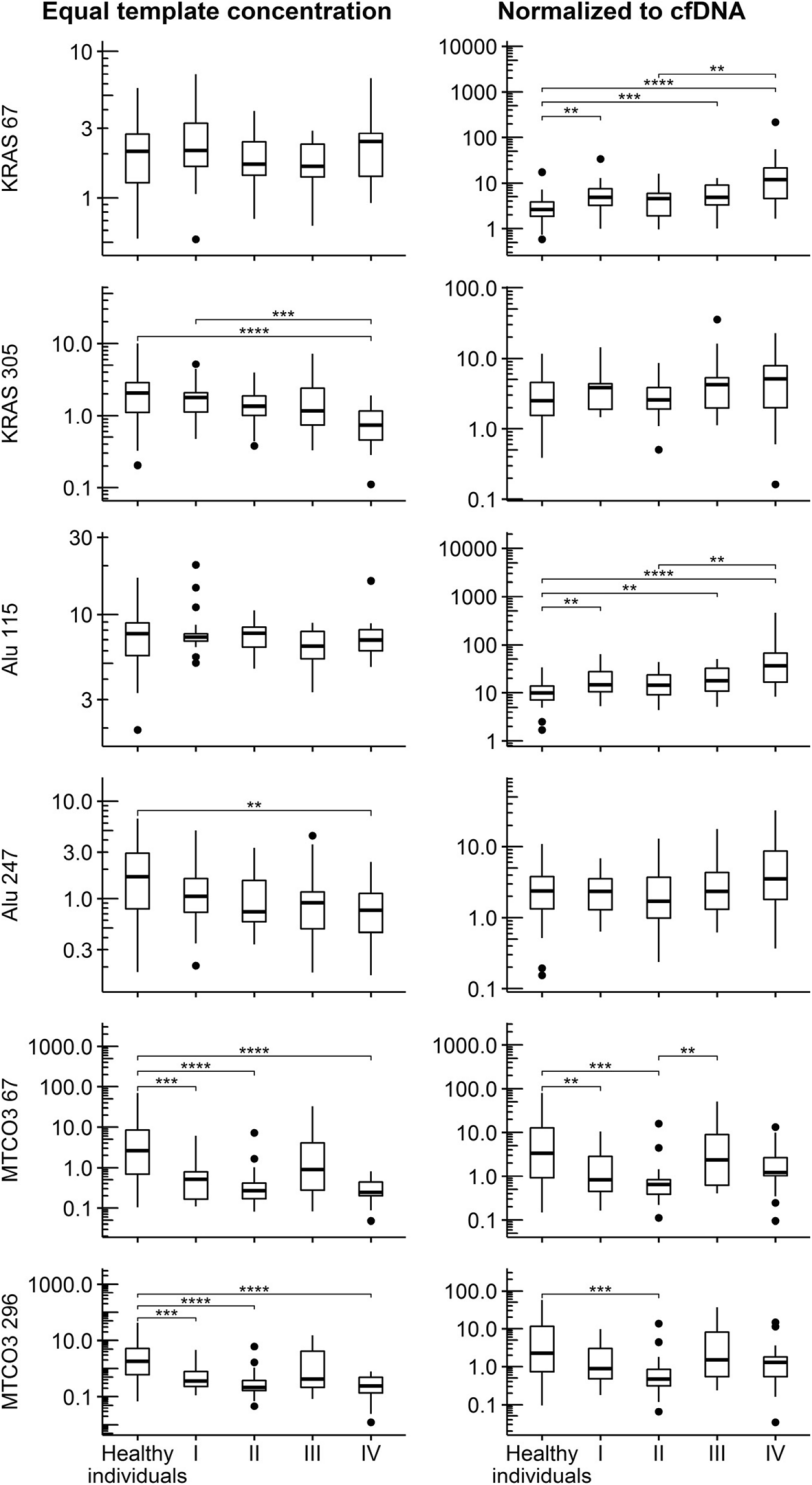
Boxplots of n-cfDNA and mt-cfDNA marker concentrations by study group, divided by histopathological UICC stage. Note. Boxplots present marker concentrations (in ng/ml) of qPCR analysis using equal template concentrations (left panel) and normalized to total cfDNA concentration (right panel). Roman numerals on x-axis ticks refer to UICC stage. Stars indicate significant group differences in multiple Mann–Whitney-Tests (Bonferroni-Holm-adjusted): * … *P* ≤ 0.05, ** … *P* ≤ 0.01, *** … *P* ≤ 0.001, **** … *P* ≤ 0.0001. UICC: Union internationale contre le cancer

To decipher cfDNA marker concentrations in relation to CRC progression, the CRC cohort was grouped by their UICC stages (Table 2 right panel and Fig. 3). Principally, the pattern of significant group differences in the ETC and NTC followed the same as described above for the comparison of healthy individuals and CRC patients. Long n-cfDNA fragments (KRAS 305 and Alu 247) differed significantly between groups in the ETC condition, while short n-cfDNA fragments (KRAS 67 and Alu 115) showed significant differences in the NTC condition. Thereby, higher UICC stages were associated with decreased long fragment concentrations in the ETC condition and increased short fragment concentrations in the NTC condition.

For the mt-cfDNA marker MTCO3 (Fig. 3, two bottom rows) similar results were obtained in both conditions, however, more significant in ETC. While higher UICC stages were generally associated with lower fragment concentrations (especially in the ETC condition), a very high variance was observed in individuals with UICC stage III, which disturbed the overall trend.

### DNA integrity indices of nuclear and mitochondrial cfDNA

**Fig. 4:**
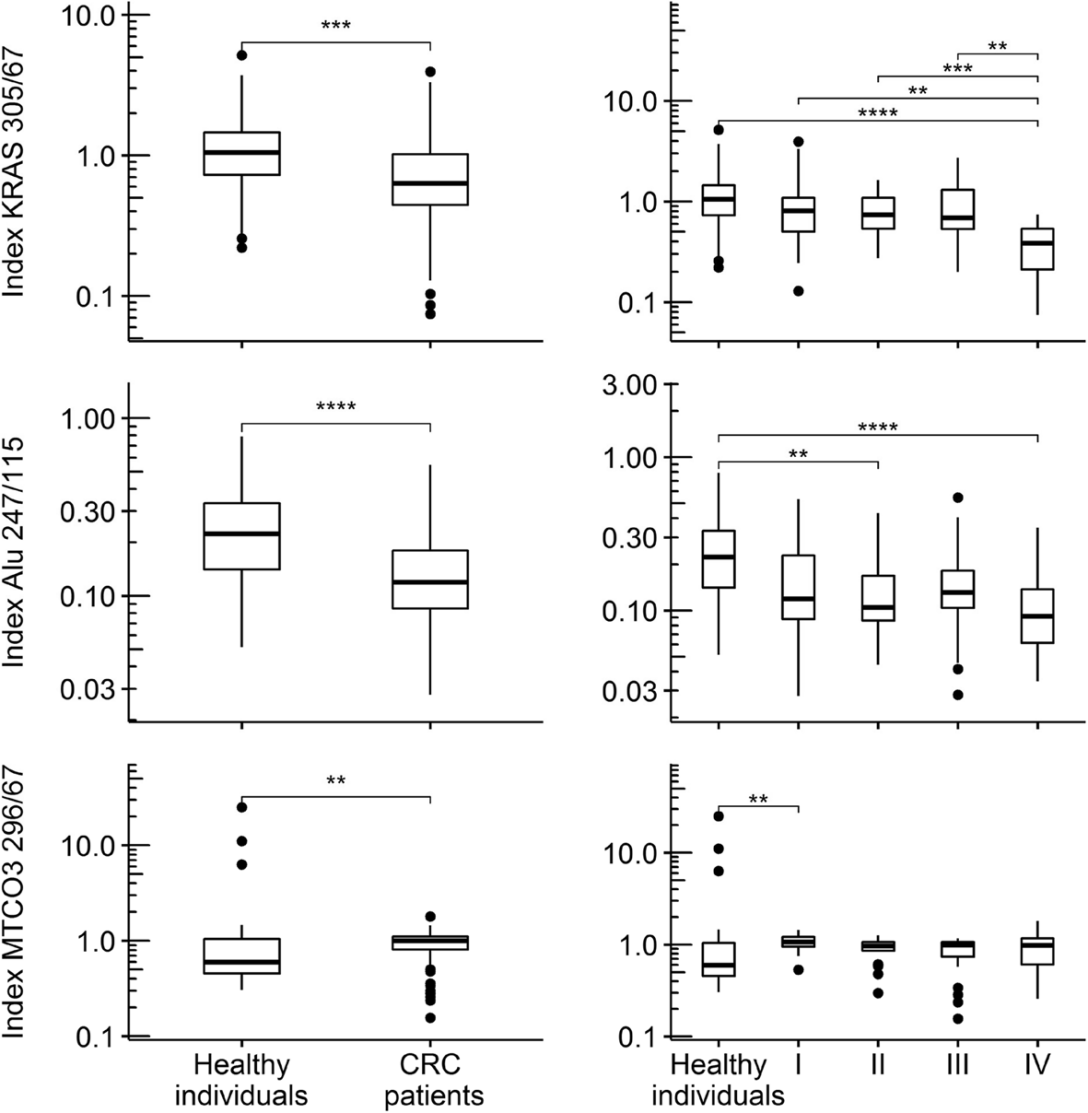
Boxplots of DNA integrity index values by study groups and UICC stage. Note. Boxplots present DII values (dimensionless) by study group (left panel) and further subdivided by UICC stage (right panel). Roman numerals on x-axis ticks refer to UICC stage. Stars indicate significant group differences in multiple Mann–Whitney-Tests (Bonferroni-Holm-adjusted in right panel): * … *P* ≤ 0.05, ** … *P* ≤ 0.01, *** … *P* ≤ 0.001, **** … *P* ≤ 0.0001. CRC: colorectal cancer; UICC: Union internationale contre le cancer

Note that the DII values are not effected of the normalization procedure and therefore are not depicted with respect to the ETC and NTC conditions. Groupwise medians (IQR’s) and non-parametric overall test results are given in Table 2 (lower third). Results of adjusted multiple groupwise comparisons are depicted in Fig. 4. In both n-cfDNA fragments lower DII’s were associated with higher UICC stages. The MTCO3 296/67 index showed no clear association with UICC stage.

### Diagnostic accuracy of individual markers

The cfDNA marker cut-offs and DII scores to differentiate between healthy individuals vs. CRC patients, healthy individuals vs. UICC stage I/II, healthy individuals vs. UICC stage III/IV, and UICC stage I/II vs. UICC stage III/IV are summarized in Supplementary Table S3. No single condition (ETC vs NTC) outperformed the other across all markers and group differences. Regarding the AUCs, in the NTC condition short n-cfDNA fragments yielded higher discriminatory power than in the ETC condition. With one exception (Alu 247 in UICC I/II vs UICC III/IV), the long n-cfDNA fragments provide better differentiation in the ETC condition. For the mt-cfDNA marker MTCO3 the analysis showed mixed results. When one of the groups to differentiate was the healthy controls, diagnostic accuracy was better in the ETC condition, but discrimination performance between UICC I/II and UICC III/IV was superior in the NTC condition.

The total cfDNA concentration yielded one of the highest AUCs in all four group differentiations ranging from 0.69 to 0.82 (all *P’*s < 0.007) and with sensitivity indices of 81% up to 88%. The specificity for all four groups was 62% (1), 57% (2), 74% (3) and 55% (4), respectively and indicates the best CRC detection rate for patients with later stadium III/IV.

### Discriminative diagnostic accuracy of predictor sets

Results of the three LASSO multinomial regression models to discriminate between healthy individuals (reference group) and persons with UICC I/II and UICC III/IV, respectively, are depicted in Table 3. Both models that based on only one predictor set (ETC or NTC, respectively) showed roughly comparable classification performance with overlapping confidence intervals of misclassification error rates. The regression model that incorporated ETC-predictors and NTC-predictors, however, showed a substantially lower misclassification error rate (33.1%) and higher multiclass AUC-value (AUC: 0.843) and outperformed each single predictor set (Table 3). Corresponding ROC curves of the models are depicted in Supplementary Figure S1. Furthermore, the misclassification error rates confidence interval of the third model, that incorporated variables of both predictor sets, does not overlap with the first two models.

**Table 3:** Estimated regression coefficients of the three relaxed multinomial LASSO models

Predictors	ETC predictor set		NTC predictor set		ETC and NTC predictor set	
	UICC I/II	UICC III/IV	UICC I/II	UICC III/IV	UICC I/II	UICC III/IV
Equal template concentration (ETC)						
KRAS 67						
KRAS 305						
Alu 115						
Alu 247	0.927	0.906			0.600	0.458
MTCO3 67						
MTCO3 296	0.947	0.955			0.894	0.923
Normalized to total cfDNA (NTC)						
KRAS 67						
KRAS 305					1.111	1.199
Alu 115			1.001	1.002		
Alu 247					1.026	1.072
MTCO3 67			0.998	0.999	0.968	0.995
MTCO3 296			0.985	0.991		
Independent						
(Intercept)	1.351	1.196	1.112	1.049	2.300	1.816
Index KRAS 305/67					0.924	0.647
Index Alu 247/115	0.374	0.348	0.224	0.196	0.691	0.768
Index MTCO3 296/67						
Total DNA ng/ml Plasma	1.000	1.003			1.008	1.033
Misclassification error	0.408 (0.379, 0.436)		0.446 (0.410, 0.482)		0.331 (0.300, 0.361)	
Multiclass AUC	0.813		0.775		0.843	

Regression coefficients are reported as relative risk ratios with healthy individuals as reference category. Predictors without coefficient values were dropped by LASSO regression procedure. Misclassification error indicates percentage (95% confidence interval) of non-correctly categorized individuals as assessed by tenfold cross validation

*UICC* Union internationale contre le cancer, ETC Equal template condition, *NTC* Normalized to Total DNA condition

## Discussion

A large number of studies have shown that higher total cfDNA levels can be detected in the plasma of colorectal cancer patients, although, especially in late tumour stages [11, 12, 35, 36]. To date, it remains unclear how the lengths of nuclear and mitochondrial cfDNA fragments contribute to total cfDNA quantity and whether they can be used as sensitive and reliable biomarkers in CRC diagnostics. It is considered that tumour-derived necrotic DNA degradation results in cfDNA fragments with a length of > 250 bp, whereas normal apoptotic cells produces fragments of around 180 bp or less [15, 37]. An increased ratio between long to small fragments (DII) was found in a significant number of studies but others did not support this theory [21–27].

Our study was designed to specifically address the question whether short and/or long fragments were altered in CRC patients compared to healthy individuals. Therefore, we intended to precisely measure n-cfDNA and mt-cfDNA independent from total concentration of isolated cfDNA, arguing that the ratio between small and long fragments in cfDNA samples from certain study groups must remain identical.

In agreement with previous findings applying fluorescent-based cfDNA quantification methods reviewed in Petit et al. [12], we detected a higher concentration of total cfDNA in plasma samples of CRC patients compared to healthy controls. In addition, the level was observed to increase with the pathological stage of CRC, suggesting an increase with tumour malignancy.

Using equal cfDNA concentration (ETC) as template for qPCR analysis, control and CRC patients showed similar levels of both KRAS 67 and Alu 115 markers. This outcome also remains unaltered in all stages of CRC, highlighting that there is no measurable difference in short n-cfDNA fragment levels between healthy and cancer group at ETC condition. However, regarding long fragments of both n-cfDNA markers KRAS 305 and Alu 247, a significant decrease was detected comparing both cohorts. Strikingly, decreased levels of long n-cfDNA fragments was also observed in later stages of CRC, although with a statistical significance only in stage IV, indicating that advanced tumour malignancy inversely contribute to n-cfDNA stability. Likewise, significantly reduced DII scores were detected for both KRAS 305/67 and Alu 247/115. Primarily, significant reductions were observed in UICC stage IV, with the most pronounced decrease of long n-cfDNA fragments. Importantly, the results changed profoundly when the qPCR data was normalized to the actual cfDNA concentration of each sample (NTC). We believe that this approach mostly resembles previous qPCR analysis, in which cfDNA samples were analysed via qPCR independent from its concentration. Accordingly, the level of short n-cfDNA fragments significantly increases in the CRC cohort and with higher pathological stages. This result demonstrates that the n-cfDNA concentration is generally higher in the plasma of CRC patients and confirmed previous findings [13, 14, 18, 20–28]. For long n-cfDNA fragments at NTC condition, the calculated quantities of both KRAS 305 as well as Alu 247 did not significantly differ between healthy individuals and all stages of CRC patients. Although, we noticed a slight increase in the level of both markers from stage II to IV, this observation was without statistical significance. At this point, our findings differed from previous studies, that reported elevated cfDNA levels including long fragmented markers [20–22, 25, 27]. Nevertheless, Mead et. al. reported increased median levels of total cfDNA and Alu 115 rising from control to benign polyps and cancer group, whereas long fragment levels of Alu (247) and Line1 (300) were comparable or even lower in CRC patients compared to individuals with benign polyps [21]. Of note, Bhangu et al. reported a significantly decreased median level of Line 297 in total CRC patients as well as patients with either stage I-III or stage IV compared to control individuals. However, in contrast to increased levels of Alu 115, no significant difference between controls and patients was found for Line 79 [26]. Furthermore, the integrity index of patients with different histopathological stages of CRC were reported to significantly decrease in stage IV compared to stage II. This observation might be explained by an increase in the level of Alu 83 that was more profound compared to that of Alu 244 [22]. A significantly decreased DII was also observed by Yörüker et al. and Sinha et al., with both research groups investigating multicopy transposable elements such as Alu in stage IV CRC patient plasma compared to healthy controls [23, 27]. Pu et al. found similar median fragment levels of Alu 219 in CRC patients with stage 0, I and II, while only patients in stage IV were found to be significantly higher compared to control individuals. In contrast, Alu 115 values were significantly higher in stage I, II and IV, thus resulting in a decreased cfDNA integrity in all stages compared to healthy controls was observed [25].

We assume that, under NTC condition, the level of especially short n-cfDNA fragments in the plasma of CRC patients increases proportional to the level of total cfDNA, particularly in advanced stages of CRC. In contrast, long n-cfDNA fragments did not, suggesting that increased levels of total cfDNA in CRC patients is not associated with raised necrotic cellular degradation. This result is also in accordance with a recent view, whereby increased tumor-derived cfDNA quantity predominantly comprises shorter fragments compared to healthy individuals [23–25]. In further support to our view, Mouliere et al. reported that the median-size distribution of cfDNA in the plasma of metastatic CRC patients were lower compared to healthy individuals utilizing Atomic Force Microscopy [38]. In this context, several recent studies applying next generation sequencing (NGS) techniques on cfDNA extracted from plasma of cancer patients revealed significantly lower levels of long fragmented cfDNA [39, 40].

The analysis of short and long fragments of MTCO3 revealed a highly significant decrease of both markers in CRC group at ETC condition. Moreover, both fragment levels were significantly reduced in almost all stages of CRC with the exception of stage III. Considering the relevance of especially early stages (I and II) for CRC detection, our data suggests an improvement for cfDNA-based diagnostics using mitochondrial markers. Of note, decreased mt-cfDNA levels were also found when normalizing our data to the total cfDNA concentration (NTC). However, this approach remarkably reduced the difference in mt-cfDNA quantity between CRC and control group as well as UICC stages, relative to the data obtained from equal template concentration. Thus, our findings may have unravelled a weak point of using unequalized total cfDNA concentrations as template in qPCR-based CRC diagnostics (NTC) and provides additional evidence for the usefulness of our approach (ETC). Surprisingly, although weak, the DII of mt-cfDNA fragments in CRC patients was significantly higher compared to healthy individuals. This observation was in contrast to the DII of n-cfDNA and may be due to significant differences between healthy individuals and CRC patients in long and short MTCO3 fragment levels. At this point, our data further suggest a fundamental difference between n-cfDNA and mt-cfDNA with regard to its integrity. However, we can only speculate whether this difference is due to an alternate origin or mode of degradation. Of note, mitochondrial DNA lacks a nucleosomal core structure and it was recently published that plasmatic mt-cfDNA is more stable compared to n-cfDNA [41]. In addition, it was reported that a substantial amount of entire cell-free mitochondria with intact respiratory metabolism are present in human plasma, next to its known presents in microvesicles [41]. Nonetheless, in our view, the prognostic value of mitochondrial DII is questionable at this point, since there is no significant difference between the UICC stages. Even so, we confirmed previous findings in which MTCO3 was used as a potential biomarker, whereby mt-cfDNA concentration in CRC patients decreases significantly compared to healthy individuals [28]. With regard to other mitochondrial target sequences, specifically MTND1, Mead et al. reported an increased mt-cfDNA level in polyp and cancer population compared to control individuals. However, in comparison to an increased Alu 115 quantity, no difference in mt-cfDNA concentration was found between poly and cancer group [21]. Strikingly, several recent studies demonstrated a significantly higher mt-cfDNA concentration (copy number) in plasma of healthy controls compared to CRC patients applying NGS-based approaches [40, 42].

Generally, we believed that the contribution of tumour-specific n- and mt-cfDNA might be far too low to explain the elevated total cfDNA level or changes in the DII in plasma of CRC patients. It is therefore necessary to consider other non-malignant cells or cellular processes as a major source of cfDNA. This is supported by research that has shown that stromal, endothelial and immune cells also constitute to the microenvironment of tumour tissue either to support or to oppose cancer formation [36, 43]. Therefore, although it is plausible that senescent tumour cells in CRC patients are frequently undergoing necrosis, cancer cell death might be covered by enhanced apoptosis from yet unknown cell origin.

An additional important objective of this study was to determine marker-specific cutoffs for the diagnostic use of biomarkers to differentiate groups. Specifically, we report the cut-offs for the following group differentiations: 1) healthy control versus total CRC patients, 2) healthy control versus CRC patients with UICC stage I and II, group 3) healthy control versus patients with stage III and IV, and group 4) UICC stage I and II versus stage III and IV. Therefore, we either used the data measured unrelated to (ETC) or dependent on the total cfDNA concentration (NTC) (Table S3). Of note, total cfDNA concentration measured spectrophotometrically yielded one of the highest AUCs in all four group differentiations and indicates the best CRC detection rate for patients with later stadium III/IV. With regard to previous studies, in which a comparable detection method was used, our results performed moderately better. For early stages (I/II), an AUC of 0.64 (*P* = 0.03) with 42% sensitivity and 75% specificity and for later stages (III/IV) an AUC of 0.63 (*P* = 0.003) with 63% sensitivity and 75% specificity was reported [44]. El-Gayar et al. distinguished CRC patients from healthy donors with an AUC of 73% (*P* = 0.004), a sensitivity of 68% and a specificity of 65% [14].

For the evaluation of diagnostic potential using single markers, best results were obtained for both mt-cfDNA fragments in the ETC condition, thus highlighting the potential of mt-cfDNA biomarkers in early stage CRC detection. This result is in agreement with data from Mead et al., in which ROC curve analysis of a single mt-cfDNA marker were able to significantly (*P* < 0.001) differentiate patients (polyps and CRC) from healthy control [21]. For single nt-cfDNA marker quantities, highest diagnostic accuracies were obtained for longer fragments of KRAS and Alu in the ETC condition and shorter fragments KRAS and Alu in the NTC condition. However, detection of CRC with high sensitivity and specificity was only reached in advanced tumour progression (UICC stages III and IV), suggesting a subordinate importance of these markers for early diagnosis. In consistency with our results, several studies reported a clear discrimination between healthy and advanced or metastatic CRC populations with a high sensitivity and specitivity of single nt-cfDNA markers with most of them targeting the Alu sequence [18, 21, 26, 27].

In our study, the DII of nt-cfDNA and mt-cfDNA proved to be effective determinants to significantly differentiate the aforementioned groups. However, we conclude that DII scores from both n- and mt-cfDNA markers in our analysis did not performed superior compared to single markers, and especially with regard to the total cfDNA concentration. In our view, this could be explained by the fact that DII determination as the ratio between long and short fragment quantities is limited by the discriminatory capability of either of its “best” single marker. For example, at ETC condition, the median quantity of Alu 247 fragments performed best, whereas in the NTC condition Alu 115 seemed to be a more reliable marker (Figs. 2 and 3).

Using LASSO multinomial logistic regression, a modern and robust statistical technique that circumvents multicollinearity problems between predictor variables that is able to identify important predictors and provide robust estimates, we investigated the relationship between biomarkers and UICC stages of colorectal cancer. We applied this modelling approach to different sets of predictor variables and evaluated the predictive accuracy of the different models using misclassification error. Both models based on a single set of biomarkers (ETC or NTC) resulted in approximately equal diagnostic prediction accuracy, whereas the model that included biomarkers of the ETC and NTC condition had approximately 30% lower misclassification error rates. Interestingly, only long fragmented biomarkers were selected as predictors in the final model, with the exception of the MTCO3 marker (67 bp).

## Conclusions

In conclusion, this study suggests that cfDNA represents a cost effective alternative to diagnose and monitor certain types of cancer including CRC. However, it is necessary to prove its reliable clinical application in settings ideally outside from case control studies. This might be achieved by utilizing quality assured biospecimen of larger population-based biobanks collected at multiple time points in the life time of individuals. A major contribution of our work to the field of research is the novel approach we applied to quantify short and long fragments of n-cfDNA and mt-cfDNA markers. Our qPCR analysis in ETC condition was conducted with equal fixed template concentrations of isolated cfDNA samples from both patients and healthy individuals, thus, following the argument that the ratio between short and long fragments should be unaltered regardless the dilution factor of cfDNA used as template. Our results suggest that the increased amounts of total cfDNA found in the bloodstream of CRC patients cannot be associated with elevated necrotic degradation processes since the quantity of large genomic cfDNA fragments were found to decrease with pathological cancer stage. Moreover, we observed reduced short and long mt-cfDNA levels, a result that presumably became less abundant if undiluted cfDNA was used as template considering that total cfDNA concentration is higher in CRC patients.

Finally, however, the limitations of the present study must also be pointed out. Due to the case–control design of the study, a systematic result-distorting effect due to differences between the study groups (e.g. age, nutrition, lifestyle etc.) cannot be ruled out. In this regard, a correlation between age and total cfDNA as well as some biomarker quantities was observed (Supplementary Tables S1, S2). These correlations seemed more pronounced in ETC compared to NTC indicating significant differences between both conditions for data evaluation. Nevertheless, a comparable median age in UICC stages and clear trend for various single biomarker quantities can be found in both ETC and NTC. Thus, it is possible that the inclusion of additional influencing factors, for example in the form of covariates in the multivariate regression model, could change the diagnostic significance of individual biomarkers. Further studies are necessary to validate the promising potential of our approach using a combined set of genomic and mitochondrial fragments to detect CRC and to differentiate between its clinicopathological stages. Moreover, it remains an open question whether our findings and prognostic models can be transferred to other types of cancer as well.

## Supplementary Information

**Additional file 1: Figure S1.** Multiclass ROC curves of the multinomial regression models discriminating between healthy individuals and patients with UICC I/II or UICC III/IV as well as between UICC I/II and UICC III/IV, respectively. The models incorporate either predictor sets in ETC or NTC condition as well as both together. **Table S1.** Spearman rank correlations (95%-CI) between age and biomarker concentrations (equal template concentrations - ETC) for complete sample and stratified by disease status, as well as UICC stage. **Table S2.** Spearman rank correlations (95%-CI) between age and biomarker concentrations (normalized to total cfDNA - NTC) for complete sample and stratified by disease status, as well as UICC stage. **Table S3.** Diagnostic cut-offs of cfDNA markers in the ETC and NTC condition between 1) Healthy individuals vs. Total CRC patients, 2) Healthy individuals vs. UICC stage I/II, 3) Healthy individuals vs. UICC stage III/IV, and 4) UICC stage I/II vs. UICC stage III/IV. **Table S4.** Oligonucleotides used in the qPCR analysis.

## Data Availability

All data generated or analysed during this study are included in this published article and its supplementary information. Original qPCR data, such as CT values, are available from the corresponding author on reasonable request.

## Abbreviations

cfDNA: Circulating Cell-free DNA
n-cfDNA: Nuclear cfDNA
mt-cfDNA: Mitochondrial cfDNA
CRC: Colorectal Cancer
ETC: Equal Template Concentrations
NTC: Normalized Template Concentrations
qPCR: Quantitative Real-Time Polymerase Chain Reaction
KRAS: Kirsten Rat Sarcoma Virus
Alu: Arthrobacter luteus
MTCO3: Mitochondrially Encoded Cytochrome C Oxidase III
DRKS: German Register for Clinical Trials
gFOBT: Guaiac-based Fecal Occult Blood Test
FIT: Fecal Immunological Test
DII: DNA Integrity Index
STARD: Standards for Reporting of Diagnostic Accuracy Studies
UICC: Union for International Cancer Control
IQR: Interquartile Range
LASSO: Least Absolute Shrinkage and Selection Operator
CT: Cycle Threshold

## References

[1] Labianca R, Nordlinger B, Beretta GD, Mosconi S, Mandalà M, Cervantes A (2013). Early colon cancer: ESMO clinical practice guidelines for diagnosis, treatment and follow-up. Ann Oncol.

[2] Davis DM, Marcet JE, Frattini JC, Prather AD, Mateka JJ, Nfonsam VN (2011). Is it time to lower the recommended screening age for colorectal cancer?. J Am Coll Surg.

[3] O'Connell JB, Maggard MA, Ko CY (2004). Colon cancer survival rates with the new American Joint Committee on Cancer sixth edition staging. J Nat Cancer Inst.

[4] McLoughlin RM, O'Morain CA (2006). Colorectal cancer screening. World J Gastroenterol.

[5] Ore L, Hagoel L, Lavi I, Rennert G (2001). Screening with faecal occult blood test (FOBT) for colorectal cancer: assessment of two methods that attempt to improve compliance. Eur J Cancer Prev.

[6] Ibañez-Sanz G, Garcia M, Milà N, Rodríguez-Moranta F, Binefa G, Gómez-Matas J (2017). False-negative rate cannot be reduced by lowering the haemoglobin concentration cut-off in colorectal cancer screening using faecal immunochemical test. Eur J Cancer Prev.

[7] Ibáñez-Sanz G, Milà N, de la Peña-Negro LC, Garcia M, Vidal C, Rodríguez-Alonso L (2021). Proton-pump inhibitors are associated with a high false-positivity rate in faecal immunochemical testing. J Gastroenterol.

[8] Herrera M, Galindo-Pumariño C, García-Barberán V, Peña C (2019). A snapshot of the tumor microenvironment in colorectal cancer: the liquid biopsy. Int J Mol Sci.

[9] Gravina S, Sedivy JM, Vijg J (2016). The dark side of circulating nucleic acids. Aging Cell.

[10] Francis G, Stein S (2015). Circulating cell-free tumour DNA in the management of cancer. Int J Mol Sci.

[11] Cervena K, Vodicka P, Vymetalkova V (2019). Diagnostic and prognostic impact of cell-free DNA in human cancers: systematic review. Mutat Res, Rev Mutat Res.

[12] Petit J, Carroll G, Gould T, Pockney P, Dun M, Scott RJ (2019). Cell-free DNA as a diagnostic blood-based biomarker for colorectal cancer: a systematic review. J Surg Res.

[13] Umetani N, Kim J, Hiramatsu S, Reber HA, Hines OJ, Bilchik AJ (2006). Increased integrity of free circulating DNA in sera of patients with colorectal or periampullary cancer: direct quantitative PCR for ALU repeats. Clin Chem.

[14] El-Gayar D, El-Abd N, Hassan N, Ali R (2016). Increased free circulating DNA integrity index as a serum biomarker in patients with colorectal carcinoma. Asian Pac J Cancer Prev.

[15] Jahr S, Hentze H, Englisch S, Hardt D, Fackelmayer FO, Hesch RD (2001). DNA fragments in the blood plasma of cancer patients: quantitations and evidence for their origin from apoptotic and necrotic cells. Can Res.

[16] Duffy MJ, Synnott NC, Crown J (2017). Mutant p53 as a target for cancer treatment. Eur J Cancer.

[17] da Silva Filho BF, Gurgel AP, Neto M, de Azevedo DA, de Freitas AC, Silva Neto Jda C (2013). Circulating cell-free DNA in serum as a biomarker of colorectal cancer. J Clin Pathol..

[18] Hao TB, Shi W, Shen XJ, Qi J, Wu XH, Wu Y (2014). Circulating cell-free DNA in serum as a biomarker for diagnosis and prognostic prediction of colorectal cancer. Br J Cancer.

[19] Leszinski G, Lehner J, Gezer U, Holdenrieder S (2014). Increased DNA integrity in colorectal cancer. In Vivo.

[20] Salem R, Ahmed R, Shaheen K, Abdalmegeed M, Hassan H (2020). DNA integrity index as a potential molecular biomarker in colorectal cancer. Egyptian J Med Human Genet.

[21] Mead R, Duku M, Bhandari P, Cree IA (2011). Circulating tumour markers can define patients with normal colons, benign polyps, and cancers. Br J Cancer.

[22] Bedin C, Enzo MV, Del Bianco P, Pucciarelli S, Nitti D, Agostini M (2017). Diagnostic and prognostic role of cell-free DNA testing for colorectal cancer patients. Int J Cancer.

[23] Yörüker EE, Özgür E, Keskin M, Dalay N, Holdenrieder S, Gezer U (2015). Assessment of circulating serum DNA integrity in colorectal cancer patients. Anticancer Res.

[24] Mouliere F, Robert B, Arnau Peyrotte E, Del Rio M, Ychou M, Molina F (2011). High fragmentation characterizes tumour-derived circulating DNA. PLoS ONE.

[25] Pu W, Xiao L, Zhou C, Zhong F, Wu Y, Gong W (2021). Cancer stage-dependent alterations in cell-free DNA in patients with colorectal cancer. J BUON.

[26] Bhangu JS, Taghizadeh H, Braunschmid T, Bachleitner-Hofmann T, Mannhalter C (2017). Circulating cell-free DNA in plasma of colorectal cancer patients - a potential biomarker for tumor burden. Surg Oncol.

[27] Sinha S, Brown H, Tabak J, Fang Z, Tertre MCD, McNamara S (2019). Multiplexed real-time polymerase chain reaction cell-free DNA assay as a potential method to monitor stage IV colorectal cancer. Surgery.

[28] Meddeb R, Dache ZAA, Thezenas S, Otandault A, Tanos R, Pastor B (2019). Quantifying circulating cell-free DNA in humans. Sci Rep.

[29] Clay Montier LL, Deng JJ, Bai Y (2009). Number matters: control of mammalian mitochondrial DNA copy number. J Genet Genom.

[30] Greaves LC, Reeve AK, Taylor RW, Turnbull DM (2012). Mitochondrial DNA and disease. J Pathol.

[31] Bossuyt PM, Reitsma JB, Bruns DE, Gatsonis CA, Glasziou PP, Irwig L (2015). STARD 2015: an updated list of essential items for reporting diagnostic accuracy studies. Clin Chem.

[32] Friedman J, Hastie T, Tibshirani R (2010). Regularization paths for generalized linear models via coordinate descent. J Stat Softw.

[33] Tibshirani R (1996). Regression shrinkage and selection Via the Lasso. J Roy Stat Soc B.

[34] Robin X, Turck N, Hainard A, Tiberti N, Lisacek F, Sanchez JC (2011). pROC: an open-source package for R and S+ to analyze and compare ROC curves. BMC Bioinformatics.

[35] Spindler KG, Boysen AK, Pallisgård N, Johansen JS, Tabernero J, Sørensen MM (2017). Cell-free DNA in Metastatic colorectal cancer: a systematic review and meta-analysis. Oncologist.

[36] Vymetalkova V, Cervena K, Bartu L, Vodicka P (2018). Circulating cell-free dna and colorectal cancer: a systematic review. Int J Mol Sci.

[37] Stroun M, Lyautey J, Lederrey C, Mulcahy HE, Anker P (2001). Alu repeat sequences are present in increased proportions compared to a unique gene in plasma/serum DNA: evidence for a preferential release from viable cells?. Ann N Y Acad Sci.

[38] Mouliere F, El Messaoudi S, Pang D, Dritschilo A, Thierry AR (2014). Multi-marker analysis of circulating cell-free DNA toward personalized medicine for colorectal cancer. Mol Oncol.

[39] Jiang P, Chan CW, Chan KC, Cheng SH, Wong J, Wong VW (2015). Lengthening and shortening of plasma DNA in hepatocellular carcinoma patients. Proc Natl Acad Sci USA.

[40] Haupts A, Vogel A, Foersch S, Hartmann M, Maderer A, Wachter N (2021). Comparative analysis of nuclear and mitochondrial DNA from tissue and liquid biopsies of colorectal cancer patients. Sci Rep.

[41] Al Amir Dache Z, Otandault A, Tanos R, Pastor B, Meddeb R, Sanchez C (2020). Blood contains circulating cell-free respiratory competent mitochondria. FASEB J.

[42] Liu Y, Zhou K, Guo S, Wang Y, Ji X, Yuan Q (2021). NGS-based accurate and efficient detection of circulating cell-free mitochondrial DNA in cancer patients. Mol Ther Nucleic Acids.

[43] Thierry AR, El Messaoudi S, Gahan PB, Anker P, Stroun M (2016). Origins, structures, and functions of circulating DNA in oncology. Cancer Metastasis Rev.

[44] Nagai Y, Sunami E, Yamamoto Y, Hata K, Okada S, Murono K (2017). LINE-1 hypomethylation status of circulating cell-free DNA in plasma as a biomarker for colorectal cancer. Oncotarget.

