# Supplementary material for: Quantification of mitochondrial cfDNA reveals new perspectives for early diagnosis of colorectal cancer
Source: BMC Cancer. 2023 Mar 30;23:291. doi: 10.1186/s12885-023-10748-y (PMC10064655; doi:10.1186/s12885-023-10748-y)
Supplement: Supplementary file 1 — Additional file 1: Figure S1. Multiclass ROC curves of the multinomial regression models discriminating between healthy individuals and patients with UICC I/II or UICC III/IV as well as between UICC I/II and UICC III/IV, respectively. The models incorporate either predictor sets in ETC or NTC condition as well as both together. Table S1. Spearman rank correlations (95%-CI) between age and biomarker concentrations (equal template concentrations - ETC) for complete sample and stratified by disease status, as well as UICC stage. Table S2. Spearman rank correlations (95%-CI) between age and biomarker concentrations (normalized to total cfDNA - NTC) for complete sample and stratified by disease status, as well as UICC stage. Table S3. Diagnostic cut-offs of cfDNA markers in the ETC and NTC condition between 1) Healthy individuals vs. Total CRC patients, 2) Healthy individuals vs. UICC stage I/II, 3) Healthy individuals vs. UICC stage III/IV, and 4) UICC stage I/II vs. UICC stage III/IV. Table S4. Oligonucleotides used in the qPCR analysis. [file 12885_2023_10748_MOESM1_ESM.docx]

**Supplementary Information:**

Multiclass ROC curves for the analysed biomarkers indicating the results of
our multinomial regression models (Figure S1). Spearman rank correlations between age and biomarker concentrations were performed for complete sample analysis at equal template concentration (ETC, Supplementary Table S1) as well as data normalized to total cfDNA concentration (NTC, Supplementary Table S2). The cfDNA marker cut-offs and DII scores to differentiate between healthy individuals vs. CRC patients, healthy individuals vs. UICC stage I/II, healthy individuals vs. UICC stage III/IV, and UICC stage I/II vs. UICC stage III/IV are summarized in Supplementary Table S3.

**Figure S1: Multiclass ROC curves of the multinomial regression models discriminating between healthy individuals and patients with UICC I/II or UICC III/IV as well as between UICC I/II and UICC III/IV, respectively. The models incorporate either predictor sets in ETC or NTC condition as well as both together.**

**
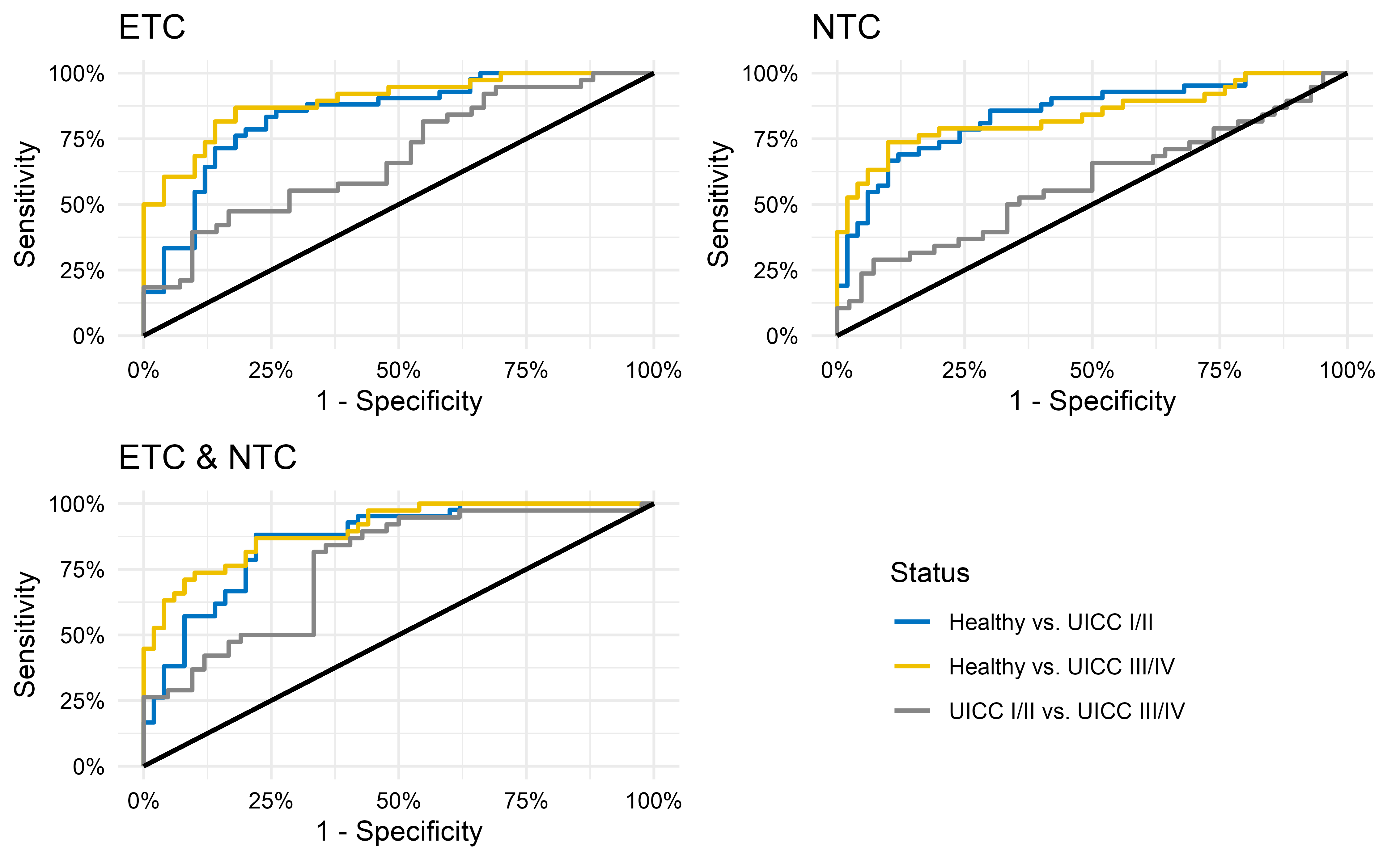
**

**Note**. UICC: Union internationale contre le cancer; ETC: Equal template condition; NTC: Normalized to Total DNA condition

**Table S1: Spearman rank correlations (95%-CI) between age and biomarker concentrations (equal template concentrations - ETC) for complete sample and stratified by disease status, as well as UICC stage.**

|  | Total | Healthy individuals | CRC patients | UICC I | UICC II | UICC III | UICC IV |
| --- | --- | --- | --- | --- | --- | --- | --- |
| Alu 247 | -0.35** (-0.50, -0.18) | -0.22 (-0.48, 0.07) | -0.26 (-0.46, -0.04) | 0.14 (-0.32, 0.55) | -0.43 (-0.73, 0.01) | -0.70* (-0.88, -0.37) | 0.13 (-0.37, 0.57) |
| Alu 115 | -0.13 (-0.30, 0.05) | -0.14 (-0.41, 0.16) | -0.09 (-0.31, 0.14) | -0.17 (-0.57, 0.30) | -0.23 (-0.61, 0.23) | -0.14 (-0.56, 0.33) | 0.00 (-0.48, 0.48) |
| Index Alu 247/115 | -0.35** (-0.49, -0.18) | -0.20 (-0.46, 0.09) | -0.23 (-0.44, -0.01) | 0.24 (-0.23, 0.62) | -0.44 (-0.74, 0.01) | -0.79** (-0.91, -0.52) | 0.10 (-0.40, 0.55) |
| KRAS 305 | -0.29* (-0.44, -0.12) | -0.20 (-0.46, 0.09) | -0.20 (-0.41, 0.02) | -0.06 (-0.49, 0.39) | -0.09 (-0.51, 0.37) | -0.74* (-0.89, -0.42) | 0.31 (-0.20, 0.69) |
| KRAS 67 | -0.05 (-0.22, 0.13) | -0.04 (-0.33, 0.25) | -0.07 (-0.29, 0.16) | 0.30 (-0.17, 0.65) | -0.29 (-0.65, 0.18) | -0.37 (-0.71, 0.10) | 0.00 (-0.48, 0.48) |
| Index KRAS 305/67 | -0.30* (-0.45, -0.13) | -0.18 (-0.44, 0.11) | -0.21 (-0.41, 0.02) | -0.17 (-0.57, 0.30) | 0.04 (-0.41, 0.48) | -0.72* (-0.89, -0.40) | 0.24 (-0.27, 0.64) |
| MTCO3 296 | -0.22 (-0.39, -0.05) | 0.29 (0.00, 0.53) | -0.19 (-0.40, 0.04) | -0.21 (-0.60, 0.25) | -0.08 (-0.50, 0.38) | -0.39 (-0.72, 0.08) | 0.04 (-0.44, 0.51) |
| MTCO3 67 | -0.28* (-0.44, -0.11) | 0.10 (-0.20, 0.37) | -0.19 (-0.40, 0.04) | -0.24 (-0.62, 0.22) | -0.16 (-0.57, 0.30) | -0.25 (-0.63, 0.23) | 0.19 (-0.32, 0.61) |
| Index MTCO3 296/67 | 0.31** (0.15, 0.47) | 0.47* (0.21, 0.67) | 0.09 (-0.14, 0.31) | 0.31 (-0.15, 0.66) | 0.29 (-0.17, 0.65) | -0.20 (-0.60, 0.28) | -0.16 (-0.60, 0.34) |
| Total cfDNA | 0.31** (0.14, 0.46) | 0.04 (-0.25, 0.32) | 0.24 (0.01, 0.44) | 0.24 (-0.22, 0.62) | 0.17 (-0.30, 0.57) | 0.44 (-0.02, 0.74) | 0.03 (-0.46, 0.50) |

**Note**. Significant correlations are marked: *** … P < .001, ** ... P < .01, * ... P < .05. CRC: colorectal cancer; UICC: Union internationale contre le cancer; ETC: Equal template condition; NTC: Normalized to Total DNA condition

**Table S2: Spearman rank correlations (95%-CI) between age and biomarker concentrations (normalized to total cfDNA - NTC) for complete sample and stratified by disease status, as well as UICC stage.**

|  | Total | Healthy individuals | CRC patients | UICC I | UICC II | UICC III | UICC IV |
| --- | --- | --- | --- | --- | --- | --- | --- |
| Alu 247 | -0.07 (-0.24, 0.11) | -0.23 (-0.48, 0.06) | -0.04 (-0.26, 0.19) | 0.34 (-0.12, 0.68) | -0.14 (-0.55, 0.33) | -0.30 (-0.66, 0.18) | 0.05 (-0.44, 0.52) |
| Alu 115 | 0.23 (0.05, 0.39) | -0.11 (-0.38, 0.18) | 0.16 (-0.07, 0.37) | 0.13 (-0.33, 0.54) | 0.07 (-0.38, 0.50) | 0.35 (-0.13, 0.69) | -0.01 (-0.48, 0.47) |
| Index Alu 247/115 | -0.35** (-0.49, -0.18) | -0.20 (-0.46, 0.09) | -0.23 (-0.44, -0.01) | 0.24 (-0.23, 0.62) | -0.44 (-0.74, 0.01) | -0.79** (-0.91, -0.52) | 0.10 (-0.40, 0.55) |
| KRAS 305 | 0.02 (-0.16, 0.19) | -0.20 (-0.46, 0.09) | 0.01 (-0.21, 0.24) | 0.23 (-0.24, 0.61) | -0.05 (-0.48, 0.40) | -0.46 (-0.75, 0.00) | 0.15 (-0.35, 0.59) |
| KRAS 67 | 0.24 (0.06, 0.40) | -0.03 (-0.31, 0.26) | 0.16 (-0.07, 0.37) | 0.36 (-0.10, 0.69) | -0.02 (-0.46, 0.43) | 0.21 (-0.27, 0.60) | 0.03 (-0.46, 0.50) |
| Index KRAS 305/67 | -0.30* (-0.45, -0.13) | -0.18 (-0.44, 0.11) | -0.21 (-0.41, 0.02) | -0.17 (-0.57, 0.30) | 0.04 (-0.41, 0.48) | -0.72* (-0.89, -0.40) | 0.24 (-0.27, 0.64) |
| MTCO3 296 | -0.09 (-0.26, 0.09) | 0.26 (-0.03, 0.51) | -0.07 (-0.29, 0.15) | 0.02 (-0.42, 0.46) | -0.02 (-0.46, 0.43) | -0.16 (-0.57, 0.32) | -0.04 (-0.51, 0.44) |
| MTCO3 67 | -0.14 (-0.31, 0.03) | 0.10 (-0.19, 0.38) | -0.06 (-0.28, 0.17) | -0.05 (-0.48, 0.40) | -0.06 (-0.49, 0.39) | -0.05 (-0.49, 0.41) | 0.07 (-0.42, 0.53) |
| Index MTCO3 296/67 | 0.31* (0.15, 0.47) | 0.47* (0.21, 0.67) | 0.09 (-0.14, 0.31) | 0.31 (-0.15, 0.66) | 0.29 (-0.17, 0.65) | -0.20 (-0.60, 0.28) | -0.16 (-0.60, 0.34) |
| Total cfDNA | 0.31* (0.14, 0.46) | 0.04 (-0.25, 0.32) | 0.24 (0.01, 0.44) | 0.24 (-0.22, 0.62) | 0.17 (-0.30, 0.57) | 0.44 (-0.02, 0.74) | 0.03 (-0.46, 0.50) |

**Note.** Significant correlations are marked: *** … P < .001, ** ... P < .01, * ... P < .05. CRC: colorectal cancer; UICC: Union internationale contre le cancer

**Table S3: Diagnostic cut-offs of cfDNA markers in the ETC and NTC condition between 1) Healthy individuals vs. Total CRC patients, 2) Healthy individuals vs. UICC stage I/II, 3) Healthy individuals vs. UICC stage III/IV, and 4) UICC stage I/II vs. UICC stage III/IV.**

| Marker | Cut-off* | AUC (95% CI) | P | Sensitivity (95% CI) | Specificity (95% CI) | PPV (95% CI) | NPV (95% CI) |
| --- | --- | --- | --- | --- | --- | --- | --- |
| 1. **Healthy individuals vs. Total CRC patients** | | | | | | | |
| **Equal template concentration** | | | | | | | |
| Total cfDNA concentration | ≥ 9.045 | 0.72 (0.64 – 0.80) | 0.007 | 0.88 (0.76 – 0.95) | 0.62 (0.51 – 0.73) | 0.59 (0.47 – 0.71) | 0.89 (0.78 – 0.96) |
| KRAS 67 | ≥ 0.922 | 0.65 (0.57 – 0.74) | 0.209 | 0.18 (0.09 – 0.31) | 0.95 (0.88 – 0.99) | 0.69 (0.39 – 0.91) | 0.65 (0.56 – 0.74) |
| KRAS 305 | ≤ 1.955 | 0.69 (0.61 – 0.77) | 0.042 | 0.54 (0.39 – 0.68) | 0.79 (0.68 – 0.87) | 0.61 (0.45 – 0.76) | 0.73 (0.63 – 0.82) |
| KRAS 305/67 | ≤ 0.935 | 0.69 (0.61 – 0.77) | 0.042 | 0.62 (0.47 – 0.75) | 0.74 (0.6 – 0.83) | 0.60 (0.45 – 0.73) | 0.76 (0.65 – 0.85) |
| Alu 115 | ≤ 8.358 | 0.68 (0.59 – 0.76) | 0.087 | 0.42 (0.28 – 0.57) | 0.84 (0.74 – 0.91) | 0.62 (0.44 – 0.78) | 0.70 (0.60 – 0.79) |
| Alu 247 | ≤ 1.29 | 0.72 (0.63 – 0.79) | 0.011 | 0.60 (0.45 – 0.74) | 0.79 (0.68 – 0.87) | 0.64 (0.49 – 0.77) | 0.76 (0.65 – 0.85) |
| Alu 247/115 | ≤ 0.186 | 0.72 (0.64 – 0.80) | 0.007 | 0.64 (0.49 – 0.77) | 0.78 (0.67 – 0.86) | 0.64 (0.49 – 0.77) | 0.78 (0.67 – 0.86) |
| MTCO3 67 | ≤ 0.44 | 0.72 (0.63 – 0.79) | 0.011 | 0.88 (0.76 – 0.95) | 0.61 (0.50 – 0.72) | 0.59 (0.47 – 0.70) | 0.89 (0.78 – 0.96) |
| MTCO3 296 | ≤ 0.494 | 0.73 (0.65 – 0.80) | 0.004 | 0.82 (0.69 – 0.91) | 0.68 (0.56 – 0.78) | 0.61 (0.49 – 0.73) | 0.86 (0.75 – 0.93) |
| MTCO3 296/67 | ≥ 0.797 | 0.72 (0.63 – 0.79) | 0.011 | 0.64 (0.49 – 0.77) | 0.76 (0.65 – 0.85) | 0.63 (0.48 – 0.76) | 0.77 (0.66 – 0.86) |
| **Normalized to total cfDNA** | | | | | | | |
| KRAS 67 | ≥ 4.183 | 0.69 (0.61 – 0.77) | 0.042 | 0.82 (0.69 – 0.91) | 0.61 (0.50, 0.72) | 0.57 (0.45, 0.69) | 0.84 (0.73, 0.93) |
| KRAS 305 | ≥ 3.814 | 0.58 (0.49, 0.66) | 0.839 | 0.72 (0.58, 0.84) | 0.49 (0.37, 0.60) | 0.47 (0.35, 0.58) | 0.74 (0.60, 0.85) |
| KRAS 305/67 | ≤ 0.935 | 0.69 (0.61 – 0.77) | 0.042 | 0.62 (0.47 – 0.75) | 0.74 (0.63 – 0.83) | 0.60 (0.45, 0.73) | 0.76 (0.65 – 0.85) |
| Alu 115 | ≥ 14.275 | 0.69 (0.61, 0.77) | 0.042 | 0.80 (0.66, 0.90) | 0.62 (0.51, 0.73) | 0.57 (0.45, 0.69) | 0.83 (0.71, 0.92) |
| Alu 247 | ≥ 3.292 | 0.52 (0.43 – 0.61) | 0.987 | 0.72 (0.58 – 0.84) | 0.40 (0.29 – 0.52) | 0.43 (0.32 – 0.54) | 0.70 (0.54 – 0.82) |
| Alu 247/115 | ≤ 0.186 | 0.72 (0.64 – 0.80) | 0.007 | 0.64 (0.49 – 0.77) | 0.78 (0.67 – 0.86) | 0.64 (0.49 – 0.77) | 0.78 (0.67 – 0.86) |
| MTCO3 67 | ≤ 1.235 | 0.64 (0.55 – 0.72) | 0.328 | 0.72 (0.58 – 0.84) | 0.59 (0.47 – 0.70) | 0.52 (0.40 – 0.64) | 0.77 (0.65 – 0.87) |
| MTCO3 296 | ≤ 1.628 | 0.66 (0.57 – 0.74) | 0.161 | 0.60 (0.45 – 0.74) | 0.70 (0.59 – 0.80) | 0.56 (0.41 – 0.69) | 0.74 (0.62 – 0.83) |
| MTCO3 296/67 | ≥ 0.797 | 0.72 (0.63 – 0.79) | 0.011 | 0.64 (0.49 – 0.77) | 0.76 (0.65 – 0.85) | 0.63 (0.48 – 0.76) | 0.77 (0.66 – 0.86) |
| 1. **Healthy individuals vs. UICC stage I+II** | | | | | | | |
| ***Equal template concentration*** | | | | | | | |
| Total cfDNA concentration | ≥ 8.55 | 0.72 (0.61, 0.81) | < .001 | 0.84 (0.71, 0.93) | 0.57 (0.41, 0.72) | 0.70 (0.57, 0.81) | 0.75 (0.57, 0.89) |
| KRAS 67 | ≥ 1.529 | 0.54 (0.44, 0.65) | 0.543 | 0.36 (0.23, 0.51) | 0.76 (0.61, 0.88) | 0.64 (0.44, 0.81) | 0.50 (0.37, 0.63) |
| KRAS 305 | ≤ 2.105 | 0.63 (0.52, 0.73) | 0.057 | 0.48 (0.34, 0.63) | 0.81 (0.66, 0.91) | 0.75 (0.57, 0.89) | 0.57 (0.43, 0.69) |
| KRAS 305/67 | ≤ 0.935 | 0.64 (0.53, 0.74) | 0.037 | 0.62 (0.47, 0.75) | 0.67 (0.50, 0.80) | 0.69 (0.53, 0.82) | 0.60 (0.44, 0.74) |
| Alu 115 | ≤ 8.358 | 0.59 (0.48, 0.69) | 0.232 | 0.42 (0.28, 0.57) | 0.79 (0.63, 0.90) | 0.70 (0.51, 0.85) | 0.53 (0.40, 0.66) |
| Alu 247 | ≤ 1.625 | 0.66 (0.56, 0.76) | 0.013 | 0.54 (0.39, 0.68) | 0.81 (0.66, 0.91) | 0.77 (0.60, 0.90) | 0.60 (0.46, 0.72) |
| Alu 247/115 | ≤ 0.119 | 0.71 (0.60, 0.80) | 0.001 | 0.82 (0.69, 0.91) | 0.57 (0.41, 0.72) | 0.69 (0.56, 0.81) | 0.73 (0.54, 0.87) |
| MTCO3 67 | ≤ 0.792 | 0.76 (0.66, 0.84) | < .001 | 0.74 (0.60, 0.85) | 0.79 (0.63, 0.90) | 0.80 (0.66, 0.91) | 0.72 (0.57, 0.84) |
| MTCO3 296 | ≤ 0.494 | 0.76 (0.66, 0.84) | < .001 | 0.82 (0.69, 0.91) | 0.69 (0.53, 0.82) | 0.76 (0.62, 0.87) | 0.76 (0.60, 0.89) |
| MTCO3 296/67 | ≥ 0.749 | 0.73 (0.63, 0.82) | < .001 | 0.60 (0.45, 0.74) | 0.88 (0.74, 0.96) | 0.86 (0.70, 0.95) | 0.65 (0.51, 0.77) |
| **Normalized to total cfDNA** | | | | | | | |
| KRAS 67 | ≥ 4.183 | 0.71 (0.60, 0.80) | 0.001 | 0.82 (0.69, 0.91) | 0.57 (0.41, 0.72) | 0.69 (0.56, 0.81) | 0.73 (0.54, 0.87) |
| KRAS 305 | ≥ 3.168 | 0.59 (0.48, 0.69) | 0.232 | 0.64 (0.49, 0.77) | 0.52 (0.36, 0.68) | 0.62 (0.47, 0.75) | 0.55 (0.38, 0.71) |
| KRAS 305/67 | ≤ 0.935 | 0.64 (0.53, 0.74) | 0.037 | 0.62 (0.47, 0.75) | 0.67 (0.50, 0.80) | 0.69 (0.53, 0.82) | 0.60 (0.44, 0.74) |
| Alu 115 | ≥ 14.275 | 0.70 (0.59, 0.79) | 0.002 | 0.80 (0.66, 0.90) | 0.57 (0.41, 0.72) | 0.69 (0.55, 0.80) | 0.71 (0.53, 0.85) |
| Alu 247 | ≤ 1.814 | 0.60 (0.49, 0.70) | 0.173 | 0.64 (0.49, 0.77) | 0.55 (0.39, 0.70) | 0.63 (0.48, 0.76) | 0.56 (0.40, 0.72) |
| Alu 247/115 | ≤ 0.119 | 0.71 (0.60, 0.80) | 0.001 | 0.82 (0.69, 0.91) | 0.57 (0.41, 0.72) | 0.69 (0.56, 0.81) | 0.73 (0.54, 0.87) |
| MTCO3 67 | ≤ 1.096 | 0.73 (0.63, 0.82) | < .001 | 0.74 (0.60, 0.85) | 0.71 (0.55, 0.84) | 0.76 (0.61, 0.87) | 0.70 (0.54, 0.83) |
| MTCO3 296 | ≤ 1.547 | 0.70 (0.59, 0.79) | 0.002 | 0.62 (0.47, 0.75) | 0.79 (0.63, 0.90) | 0.78 (0.62, 0.89) | 0.63 (0.49, 0.76) |
| MTCO3 296/67 | ≥ 0.749 | 0.73 (0.63, 0.82) | < .001 | 0.60 (0.45, 0.74) | 0.88 (0.74, 0.96) | 0.86 (0.70, 0.95) | 0.65 (0.51, 0.77) |
| 1. **Healthy individuals vs. UICC stage III+IV** | | | | | | | |
| **Equal template concentration** | | | | | | | |
| Total cfDNA concentration | ≥ 9.135 | 0.82 (0.72, 0.89) | < .001 | 0.88 (0.76, 0.95) | 0.74 (0.57, 0.87) | 0.81 (0.69, 0.91) | 0.82 (0.65, 0.93) |
| KRAS 67 | ≥ 0.922 | 0.52 (0.41, 0.63) | 0.834 | 0.18 (0.09, 0.31) | 0.97 (0.86, 1.00) | 0.90 (0.55, 1.00) | 0.47 (0.36, 0.59) |
| KRAS 305 | ≤ 1.395 | 0.70 (0.60, 0.80) | 0.006 | 0.66 (0.51, 0.79) | 0.76 (0.60, 0.89) | 0.79 (0.63, 0.90) | 0.63 (0.48, 0.77) |
| KRAS 305/67 | ≤ 0.743 | 0.74 (0.63, 0.83) | < .001 | 0.74 (0.60, 0.85) | 0.74 (0.57, 0.87) | 0.79 (0.64, 0.89) | 0.68 (0.52, 0.82) |
| Alu 115 | ≤ 8.198 | 0.62 (0.52, 0.73) | 0.166 | 0.42 (0.28, 0.57) | 0.89 (0.75, 0.97) | 0.84 (0.64, 0.95) | 0.54 (0.41, 0.67) |
| Alu 247 | ≤ 1.29 | 0.72 (0.61, 0.81) | 0.003 | 0.60 (0.45, 0.74) | 0.87 (0.72, 0.96) | 0.86 (0.70, 0.95) | 0.62 (0.48, 0.75) |
| Alu 247/115 | ≤ 0.186 | 0.73 (0.62, 0.82) | 0.002 | 0.64 (0.49, 0.77) | 0.84 (0.69, 0.94) | 0.84 (0.69, 0.94) | 0.64 (0.49, 0.77) |
| MTCO3 67 | ≤ 0.44 | 0.76 (0.66, 0.85) | < .001 | 0.88 (0.76, 0.95) | 0.61 (0.43, 0.76) | 0.75 (0.62, 0.85) | 0.79 (0.60, 0.92) |
| MTCO3 296 | ≤ 0.494 | 0.75 (0.65, 0.84) | < .001 | 0.82 (0.69, 0.91) | 0.66 (0.49, 0.80) | 0.76 (0.62, 0.87) | 0.74 (0.56, 0.87) |
| MTCO3 296/67 | ≥ 0.797 | 0.66 (0.55, 0.76) | 0.052 | 0.64 (0.49, 0.77) | 0.68 (0.51, 0.82) | 0.73 (0.57, 0.85) | 0.59 (0.43, 0.74) |
| **Normalized to total cfDNA** | | | | | | | |
| KRAS 67 | ≥ 6.548 | 0.80 (0.70, 0.87) | < .001 | 0.96 (0.86, 1.00) | 0.58 (0.41, 0.74) | 0.75 (0.63, 0.85) | 0.92 (0.73, 0.99) |
| KRAS 305 | ≥ 4.349 | 0.66 (0.55, 0.76) | 0.052 | 0.74 (0.60, 0.85) | 0.55 (0.38, 0.71) | 0.69 (0.54, 0.80) | 0.62 (0.44, 0.78) |
| KRAS 305/67 | ≤ 0.743 | 0.74 (0.63, 0.83) | < .001 | 0.74 (0.60, 0.85) | 0.74 (0.57, 0.87) | 0.79 (0.64, 0.89) | 0.68 (0.52, 0.82) |
| Alu 115 | ≥ 19.874 | 0.77 (0.67, 0.86) | < .001 | 0.92 (0.81, 0.98) | 0.58 (0.41, 0.74) | 0.74 (0.62, 0.84) | 0.85 (0.65, 0.96) |
| Alu 247 | ≥ 3.417 | 0.61 (0.50, 0.72) | 0.226 | 0.72 (0.58, 0.84) | 0.47 (0.31, 0.64) | 0.64 (0.50, 0.77) | 0.56 (0.38, 0.74) |
| Alu 247/115 | ≤ 0.186 | 0.73 (0.62, 0.82) | 0.002 | 0.64 (0.49, 0.77) | 0.84 (0.69, 0.94) | 0.84 (0.69, 0.94) | 0.64 (0.49, 0.77) |
| MTCO3 67 | ≤ 3.868 | 0.59 (0.48, 0.69) | 0.375 | 0.46 (0.32, 0.61) | 0.76 (0.60, 0.89) | 0.72 (0.53, 0.86) | 0.52 (0.38, 0.65) |
| MTCO3 296 | ≤ 1.628 | 0.60 (0.49, 0.71) | 0.297 | 0.60 (0.45, 0.74) | 0.61 (0.43, 0.76) | 0.67 (0.51, 0.80) | 0.53 (0.38, 0.69) |
| MTCO3 296/67 | ≥ 0.797 | 0.66 (0.55, 0.76) | 0.052 | 0.64 (0.49, 0.77) | 0.68 (0.51, 0.82) | 0.73 (0.57, 0.85) | 0.59 (0.43, 0.74) |
| 1. **UICC stage I+II + UICC stage III+IV** | | | | | | | |
| **Equal template concentration** | | | | | | | |
| Total cfDNA concentration | ≥ 16.245 | 0.69 (0.57, 0.79) | 0.002 | 0.81 (0.66, 0.91) | 0.55 (0.38, 0.71) | 0.67 (0.52, 0.79) | 0.72 (0.53, 0.87) |
| KRAS 67 | ≤ 1.504 | 0.59 (0.47, 0.70) | 0.157 | 0.76 (0.61, 0.88) | 0.39 (0.24, 0.57) | 0.58 (0.44, 0.71) | 0.60 (0.39, 0.79) |
| KRAS 305 | ≤ 0.873 | 0.69 (0.57, 0.79) | 0.002 | 0.88 (0.74, 0.96) | 0.47 (0.31, 0.64) | 0.65 (0.51, 0.77) | 0.78 (0.56, 0.93) |
| KRAS 305/67 | ≤ 0.696 | 0.64 (0.52, 0.74) | 0.028 | 0.57 (0.41, 0.72) | 0.71 (0.54, 0.85) | 0.69 (0.51, 0.83) | 0.60 (0.44, 0.74) |
| Alu 115 | ≤ 6.221 | 0.65 (0.54, 0.75) | 0.016 | 0.83 (0.69, 0.93) | 0.45 (0.29, 0.62) | 0.62 (0.49, 0.75) | 0.71 (0.49, 0.87) |
| Alu 247 | ≤ 1.29 | 0.56 (0.45, 0.67) | 0.288 | 0.29 (0.16, 0.45) | 0.87 (0.72, 0.96) | 0.71 (0.44, 0.90) | 0.52 (0.39, 0.65) |
| Alu 247/115 | ≤ 0.214 | 0.55 (0.43, 0.66) | 0.369 | 0.26 (0.14, 0.42) | 0.87 (0.72, 0.96) | 0.69 (0.41, 0.89) | 0.52 (0.39, 0.64) |
| MTCO3 67 | ≥ 0.198 | 0.56 (0.45, 0.67) | 0.288 | 0.33 (0.20, 0.50) | 0.82 (0.66, 0.92) | 0.67 (0.43, 0.85) | 0.53 (0.39, 0.66) |
| MTCO3 296 | ≥ 0.4 | 0.57 (0.46, 0.68) | 0.217 | 0.64 (0.48, 0.78) | 0.50 (0.33, 0.67) | 0.59 (0.43, 0.73) | 0.56 (0.38, 0.73) |
| MTCO3 296/67 | ≤ 0.855 | 0.61 (0.50, 0.72) | 0.072 | 0.81 (0.66, 0.91) | 0.39 (0.24, 0.57) | 0.60 (0.46, 0.72) | 0.65 (0.43, 0.84) |
| **Normalized to total cfDNA** | | | | | | | |
| KRAS 67 | ≥ 6.548 | 0.68 (0.56, 0.78) | 0.005 | 0.76 (0.61, 0.88) | 0.58 (0.41, 0.74) | 0.67 (0.52, 0.80) | 0.69 (0.50, 0.84) |
| KRAS 305 | ≥ 4.349 | 0.68 (0.56, 0.78) | 0.005 | 0.79 (0.63, 0.90) | 0.55 (0.38, 0.71) | 0.66 (0.51, 0.79) | 0.70 (0.51, 0.85) |
| KRAS 305/67 | ≤ 0.696 | 0.64 (0.52, 0.74) | 0.028 | 0.57 (0.41, 0.72) | 0.71 (0.54, 0.85) | 0.69 (0.51, 0.83) | 0.60 (0.44, 0.74) |
| Alu 115 | ≥ 28.863 | 0.65 (0.54, 0.75) | 0.016 | 0.81 (0.66, 0.91) | 0.47 (0.31, 0.64) | 0.63 (0.49, 0.76) | 0.69 (0.48, 0.86) |
| Alu 247 | ≥ 1.741 | 0.60 (0.48, 0.71) | 0.109 | 0.50 (0.34, 0.66) | 0.71 (0.54, 0.85) | 0.66 (0.47, 0.81) | 0.56 (0.41, 0.71) |
| Alu 247/115 | ≤ 0.214 | 0.55 (0.43, 0.66) | 0.369 | 0.26 (0.14, 0.42) | 0.87 (0.72, 0.96) | 0.69 (0.41, 0.89) | 0.52 (0.39, 0.64) |
| MTCO3 67 | ≥ 0.942 | 0.72 (0.61, 0.82) | < .001 | 0.69 (0.53, 0.82) | 0.76 (0.60, 0.89) | 0.76 (0.60, 0.89) | 0.69 (0.53, 0.82) |
| MTCO3 296 | ≥ 0.995 | 0.65 (0.54, 0.75) | 0.016 | 0.67 (0.50, 0.80) | 0.63 (0.46, 0.78) | 0.67 (0.50, 0.80) | 0.63 (0.46, 0.78) |
| MTCO3 296/67 | ≤ 0.855 | 0.61 (0.50, 0.72) | 0.072 | 0.81 (0.66, 0.91) | 0.39 (0.24, 0.57) | 0.60 (0.46, 0.72) | 0.65 (0.43, 0.84) |

**Note.** * Cutoff-value indicate assignment to 2nd group. UICC: Union internationale contre le cancer; CRC: colorectal cancer; AUC: Area under curve; P values from Wilcoxon-Mann-Whitney U-test; CI: confidence interval; PPV: positive predictive value; NPV: negative predictive value; ETC: Equal template condition; NTC: Normalized to Total DNA condition

**Table S4: Oligonucleotides used in the qPCR analysis.**

| Oligonucleotide | Sequence 5′ → 3′ | Amplicon size (bp) |
| --- | --- | --- |
| KRAS Fwd1 | CCTTGGGTTTCAAGTTATATG | 67 |
| KRAS Fwd2 | GCCTGCTGAAAATGACTGA | 305 |
| KRAS Rev1 | CCCTGACATACTCCCAAGGA |  |
| Alu Fwd1 | CCTGAGGTCAGGAGTTCGAG | 115 |
| Alu Rev1 | CCCGAGTAGCTGGGATTACA |  |
| Alu Fwd2 | GTGGCTCACGCCTGTAATC | 247 |
| Alu Rev2 | CAGGCTGGAGTGCAGTGG |  |
| MTCO3 Fwd | GACCCACCAATCACATGC |  |
| MTCO3 Rev1 | TGAGAGGGCCCCTGTTAG | 67 |
| MTCO3 Rev2 | CTCAGAAAAATCCTGCGAAGA | 296 |
